# Visual adaptation reveals multichannel coding for numerosity

**DOI:** 10.3389/fpsyg.2023.1125925

**Published:** 2023-04-24

**Authors:** Lauren S. Aulet, Stella F. Lourenco

**Affiliations:** ^1^Department of Psychology, Carnegie Mellon University, Pittsburgh, PA, United States; ^2^Department of Psychology, Emory University, Atlanta, GA, United States

**Keywords:** number perception, numerosity, adaptation, vision, parietal cortex

## Abstract

Visual numerosity is represented automatically and rapidly, but much remains unknown about the computations underlying this perceptual experience. For example, it is unclear whether numerosity is represented with an opponent channel or multichannel coding system. Within an opponent channel system, all numerical values are represented *via* the relative activity of two pools of neurons (i.e., one pool with a preference for small numerical values and one pool with a preference for large numerical values). However, within a multichannel coding system, all numerical values are represented directly, with separate pools of neurons for each (discriminable) numerical value. Using an adaptation paradigm, we assessed whether the visual perception of number is better characterized by an opponent channel or multichannel system. Critically, these systems make distinct predictions regarding the pattern of aftereffects exhibited when an observer is adapted to an intermediate numerical value. Opponent channel coding predicts no aftereffects because both pools of neurons adapt equally. By contrast, multichannel coding predicts repulsive aftereffects, wherein numerical values smaller than the adapter are underestimated and those larger than the adapter are overestimated. Consistent with multichannel coding, visual adaptation to an intermediate value (50 dots) yielded repulsive aftereffects, such that participants underestimated stimuli ranging from 10–50 dots, but overestimated stimuli ranging from 50–250 dots. These findings provide novel evidence that the visual perception of number is supported by a multichannel, not opponent channel, coding system, and raise important questions regarding the contributions of different cortical regions, such as the ventral and lateral intraparietal areas, to the representation of number.

## Introduction

Extensive research, across species and development, has provided robust evidence for a non-verbal, perceptual system that encodes the numerosity of a set (Strauss and Curtis, 1981; Cantlon and Brannon, 2007; Lourenco et al., 2012; Aulet et al., 2019). In this system, number is estimated rapidly and approximately, without counting. One key characteristic of number perception is that it is ratio-dependent (Gallistel and Gelman, 1992; Dehaene, 2011). Specifically, the ability to discriminate stimuli by number follows Weber’s law, such that as the ratio (i.e., smaller number / larger number) between numerical values increases, discrimination accuracy decreases (Dehaene, 2003; Halberda and Feigenson, 2008).

Another key characteristic of number perception is that, like other perceptual attributes (Clifford and Rhodes, 2005), it is susceptible to adaptation (Burr and Ross, 2008). For example, after prolonged exposure to visual dot arrays of relatively small numerical value (e.g., 30 dots), participants overestimate the number of dots in subsequently presented arrays. Likewise, after prolonged exposure to visual dot arrays of large numerical value (e.g., 400 dots), participants underestimate the number of dots in subsequently presented arrays. Because susceptibility to adaptation is considered a hallmark of perceptual, as opposed to cognitive, processing, the demonstration of number adaptation by Burr and Ross (2008) provided support for the hypothesis that number is a genuine perceptual dimension (see also, Anobile et al., 2016).

Researchers have leveraged number adaptation to shed light on the neural and computational mechanisms underlying number perception. For example, Burr and colleagues (Arrighi et al., 2014; Aagten-Murphy and Burr, 2016) found that number adaptation aftereffects are not retinotopic, as originally thought (Dehaene, 2009). Instead, these aftereffects are spatiotopic, such that they are anchored to a particular location in external space (e.g., a particular location on screen), not a particular location on the retina (Viswanathan and Nieder, 2020; Togoli et al., 2021). Dimensions that exhibit spatiotopic adaptation are typically high-level visual dimensions, processed relatively late in the visual stream, compared to low-level visual dimensions, processed relatively early in the visual stream (Knapen et al., 2010; Zimmermann et al., 2016). Although number may be initially encoded in early visual cortex (Park et al., 2016), recent research corroborated the claim that number *adaptation*, specifically, is a relatively high-level visual phenomenon, showing that number adaptation occurs across modalities (e.g., vision and audition), across format (e.g., sequential and simultaneous presentation; Anobile et al., 2016), and is modulated by attention (Castaldi et al., 2019; Cai et al., 2022).

Despite advances in our understanding of number perception, much is still unknown about the computations that give rise to the adaptation aftereffects and how number is perceived by the visual system. Work on perceptual adaptation, more generally, suggests that adaptation aftereffects can result from two distinct coding schemes: opponent channel or multichannel (Suzuki, 2005; Webster, 2015). For dimensions best described by opponent channel coding (e.g., color [red-green]; Hurvich and Jameson, 1957), each dimensional value is represented by the combined activation of only two channels [red and green], each with a preferred tuning to the most extreme values at either end of the dimension. For example, equal activation of both the red and green channels results in a gray percept. By contrast, for dimensions best described by multichannel coding (e.g., spatial frequency; Blakemore and Sutton, 1969), each dimension is represented by different channels, each with a preferred tuning at a specific value.

Critically, opponent and multichannel systems typically yield different patterns of perceptual aftereffects, referred to as renormalization and repulsion, respectively (Storrs and Arnold, 2015). Renormalization aftereffects are unidirectional, such that after adaptation, perception of the adapted dimension is uniformly biased toward the less-adapted channel. For example, after adaptation to a red stimulus, all subsequently presented stimuli appear less red (i.e., more green), regardless of whether these stimuli are more or less red than the initial adaptor. Several aspects of face perception, such as face emotion, also exhibit renormalization (Webster and MacLeod, 2011). Adaptation to a slightly angry face results in subsequently presented face stimuli appearing less angry, regardless of whether the stimuli are more or less angry than the initial adaptor (Skinner and Benton, 2010; Rhodes et al., 2017).

By contrast, repulsive aftereffects are bidirectional, such that after adaptation, perception of the adapted dimension is biased away (i.e., repulsed) from the value of the adaptor. For example, after adaptation to stimuli of a particular size, stimuli smaller in size than the adaptor are underestimated, and stimuli larger in size than the adaptor are overestimated (Blakemore and Sutton, 1969; Storrs and Arnold, 2017). Likewise, adaptation to gaze direction exhibits repulsive aftereffects (Calder et al., 2008; Lawson et al., 2011), such that after adaptation to a 10° leftward gaze, leftward gazes greater than 10° are perceived as even more leftward, and leftward gazes less than 10° are perceived as less leftward (i.e., more direct).

To date, there has been little work explicitly evaluating whether number adaptation exhibits opponent or multichannel coding. On the one hand, some researchers have suggested that number adaptation is consistent with opponent channel coding (Dehaene, 2009). Most recently, Schwiedrzik et al. (2016) suggested that the cross-adaptation observed between motion direction and numerosity in their study reflected characteristics of opponent channel coding. The primary evidence put forth in favor of this claim was that the adaptation aftereffects occurred even when the distance between the adaptor and test values were very large. This is consistent with opponent channel coding, which predicts that the strength of the aftereffect should increase as distance between the adaptor and test value increases, whereas for multichannel coding, the strength of the aftereffect should decrease as distance between the adaptor and test value increases (Webster, 2011).

In contrast with Schwiedrzik et al. (2016), other work has suggested that number may be encoded according to multichannel coding (Tsouli et al., 2019). For example, a comparison of results across studies suggests that number adaptation exhibits repulsive aftereffects. That is, in one study, adaptation to a particular value (e.g., 20) resulted in underestimation of stimuli larger than 20 (Tsouli et al., 2019), and in another study, adaptation to that value resulted in overestimation of stimuli smaller than 20 (Fornaciai et al., 2016).

Differences in stimuli and procedures across studies make it difficult to conclusively determine whether number aftereffects are consistent with either opponent channel or multichannel coding. In particular, number adaptation is most commonly measured using a magnitude comparison task, where participants compare the numerical value of a stimulus presented in an adapted location (i.e., the “test” stimulus) and the numerical value of a stimulus presented in a non-adapted location (i.e., the “probe” stimulus). For example, following adaptation to a large value (e.g., 400), participants judged test stimuli to be smaller than probe stimuli more often than they did when not adapted (Burr and Ross, 2008). In this kind of task, it is common to hold the numerical value of either the test or the probe stimulus constant (Fornaciai et al., 2016; Schwiedrzik et al., 2016). As a result of this procedure, the ability to measure adaptation aftereffects is confounded with the difficulty of number discrimination. Thus, in order to measure aftereffects in an unbiased manner, stimulus values should be equally variable in the adapted location (test stimulus) and non-adapted location (probe stimulus), respectively.

Moreover, previous work on number adaptation has typically focused on adaptation to very small or very large values. For example, as described earlier, Burr and Ross (2008) found that that adaptation to a small value (e.g., 30) induced overestimation whereas adaptation to a large value (e.g., 400) induced underestimation. However, although this is often referred to as a repulsive aftereffect, this does not consistute evidence for “bidirectional” aftereffects as defined in the present work. Here, unidirectional and bidirectional aftereffects refer to the effect of a single adaptation value on all possible test values. In other words, unidirectional aftereffects occur when adaptation to a specific value results in either over or underestimation across all test values, regardless of whether those values are smaller or larger than that of the adaptor value. By contrast, bidirectional aftereffects occur when adaptation to a specific value results in underestimation of values smaller than the adaptor value and overestimation of values larger than the adaptor value. However, when extreme adaptor values (i.e., the endpoints of the stimulus range) are used, it is not possible to examine the effect of adaptation on values smaller and values larger than the adaptor value. As a result, such a paradigm cannot distinguish between unidirectional and bidirectional aftereffects, and therefore, cannot distinguish between opponent channel and multichannel coding schemes.

Several behavioral paradigms, most commonly used to study face perception, have been developed to more definitively distinguish opponent and multichannel coding (Calder et al., 2008). For example, if gaze direction is coded *via* an opponent channel system, then adaptation to a “neutral” (middle; e.g., center gaze) value will result in no adaptation aftereffects, because the two opponent channels (e.g., maximally leftward and maximally rightward gaze) exhibit equal reduction in activation. Specifically, because the relative adaptation is equal in both channels, the response to the average or “neutral” value (center gaze), which is represented as the location of intersection of opponent channel firing, remains unchanged. Conversely, if gaze direction is encoded according to a multichannel coding scheme, with a dedicated channel for representing center gaze, then adaptation to a “neutral” value (center gaze), results in repulsive (i.e., bidirectional) aftereffects, such that subsequently presented stimuli are perceived as more extreme. In other words, a slightly leftward gaze appears more leftward and a slightly rightward gaze appears more rightward. Calder et al. (2008) found that adaptation to direct gaze resulted in visual aftereffects in which participants perceived slightly leftward/rightward gaze as significantly more leftward/rightward (i.e., less direct), suggesting that gaze direction is encoded according to a multichannel coding scheme with at least three channels: left, direct (center), and right gaze.

**Figure 1:**
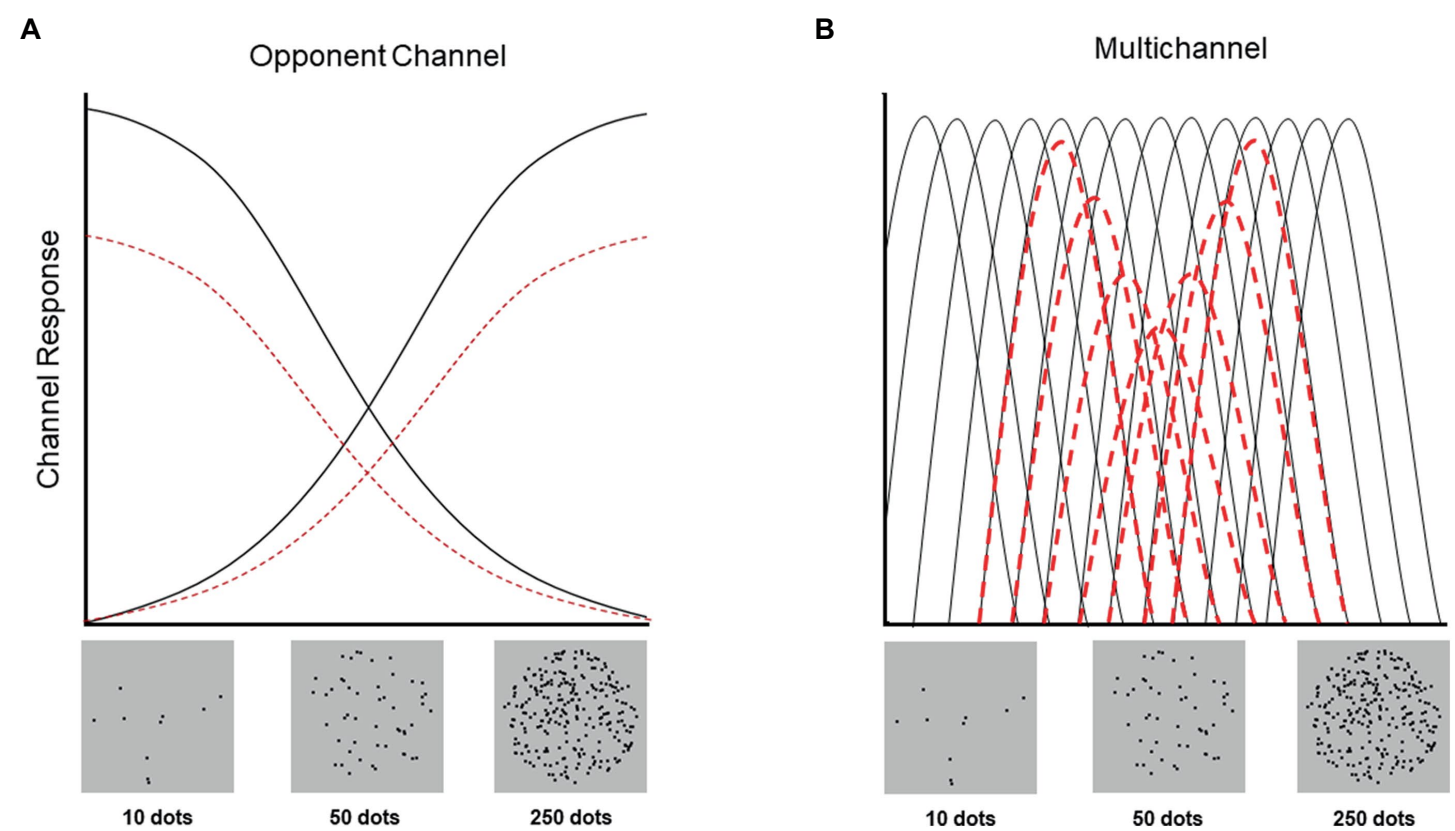
Schematic depiction of adaption to an intermediate value (i.e., 50 dots) in opponent channel and multichannel coding systems where the numerical values represented by this system is hypothesized to range from 10 to 250 (Anobile et al., 2016). The y-axis refers to the intensity of each channel’s response and the x-axis refers to the numerical value of the stimulus presented (without adaptation [black] and with adaptation to 50 [red]). **(A)** In an opponent channel system, adaptation to 50 does not result in a perceptual aftereffect because both channels (small and large numerosities) are adapted equally. In other words, although both small and large channels respond less strongly following adaptation (red dashed lines), no perceptual aftereffects occur because stimuli are represented by the difference in response between the two channels, which remains constant (depicted by the location of the intersection of the two solid black lines and two dashed red lines, on the horizontal axis). **(B)** By contrast, in a multichannel system, adaptation to 50 (or any other value) results in repulsive perceptual aftereffects, with the strongest effects occurring around the adapted value (depicted by the relative difference in height between the solid black and dashed red lines). For example, if viewing 40 dots normally (i.e., in the absence of adaptation) results in channels tuned to 40 responding at their maximum intensity, and channels tuned to 30 and 50 responding at 50% their maximum intensity, then following adaptation to 50 dots, viewing 40 dots may result in channels tuned to 50 responding only at 25% their maximum intensity. Because of this reduction, 40 dots will be perceived more similar to 35 dots.

In the present study, we capitalized on the capacity of behavioral paradigms to distinguish neural coding schemes to assess whether the behavioral signatures of numerosity perception are more consistent with opponent channel or multichannel neural coding. We examined adaptation aftereffects across two ranges of numerosity values (“small” condition: 10–50 dots; “large” condition: 50–250 dots), following adaptation to a “neutral”/central numerical value (50, the geometric mean of the total stimulus range). Specifically, we hypothesized that when participants are adapted to a neutral value, if number is encoded in an opponent channel fashion, participants should not exhibit adaptation aftereffects (Figure 1A). Crucially, even if 50 does not represent the exact center or ‘neutral’ value in the number dimension, if numerosity is encoded according to an opponent channel system, then participants should nonetheless exhibit unidirectional adaptation effects, such that all stimuli should be either underestimated or overestimated. By contrast, if visual numerosity is encoded in a multichannel fashion, participants should exhibit bidirectional adaptation, such that numbers smaller than 50 are underestimated and numbers larger than 50 are overestimated (Figure 1B).

## Method

### Participants

Eight adults (*n* = 4/stimulus condition; *M*_age_ = 19.67 years, 4 male and 4 female) participated in this experiment for course credit. All participants had normal or corrected-to-normal vision. Procedures were approved by the Institutional Review Board (IRB) at Emory University.

### Stimuli

Stimuli were dot arrays, designed in accordance with those used by Burr and Ross (2008). Dot arrays were comprised of black and white elements (50% each), presented on a gray background (400 × 400 px) and were generated with a custom Python script. Stimuli were dot arrays with elements of constant size (4 × 4 px), randomly arranged within a constant field area (300 × 300 px). The number of elements in the arrays ranged from 10 to 250 (“small” condition: 10, 15, 20, 25, 30, 35, 40, 45, 50; “large” condition: 50, 75, 100, 125, 150, 175, 200, 225, 250). This range was chosen based on previous work suggesting that the approximate number system extends across this range (Anobile et al., 2016; Pomè et al., 2019). Stimulus values within these ranges were chosen in order to equate ratio differences between stimuli across small and large conditions. There were 20 exemplars of each numerosity, varying only in the placement of the elements, and exemplars were randomly selected.

### Procedure

Tasks were created in Psychopy (Peirce et al., 2019) and presented on a desktop computer with a 19-inch screen (1,280 × 1,024 px). Participants were seated in a chinrest approximately 40 cm from the monitor. All participants completed both the control and adaptation tasks, allowing for within-subject assessment of the adaptation effect. All participants completed the control task first and adaptation task second, in order to avoid carryover adaptation effects. Control and adaptation tasks were identical except for the inclusion of the adaptation and top-up adaptation phases in the adaptation task. Within each task, participants were presented with four blocks of trials, consisting of 60 trials each. In two blocks, participants were presented with stimuli to the left of fixation, and in the other two blocks, participants were presented with stimuli to the right of fixation (order counterbalanced across participants; Burr and Ross, 2008). Thus, all participants completed 240 control trials and 240 adaptation trials.

At the beginning of the adaptation task, participants completed the adaptation phase. During this phase, participants were presented 16 dot arrays of constant numerosity (50 dots), but varying in position, for 250 ms each (Aagten-Murphy and Burr, 2016). These adaptor stimuli were presented 7° away from fixation (which was presented centrally), to the top left, or bottom right. Participants then completed the test phase. During this phase, top-up adaptation occurred at the start of each trial; top-up adaptation was identical to the adaptation phase except that participants viewed eight arrays as opposed to 16. Following top-up adaptation (500 ms ISI), test stimuli were displayed in the same position as the adaptor for 500 ms, and then the probe stimulus was displayed for 500 ms, directly above or below the test stimulus. Using the keypad, participants indicated whether the top or bottom stimulus was more numerous. All numbers were used for test and probe stimuli, such that all numbers were compared to every other (small condition: 10, 15, 20, 25, 30, 35, 40, 45, 50; large condition: 50, 75, 100, 125, 150, 175, 200, 225, 250), when in the adapted and non-adapted locations.

## Results

**Figure 2:**
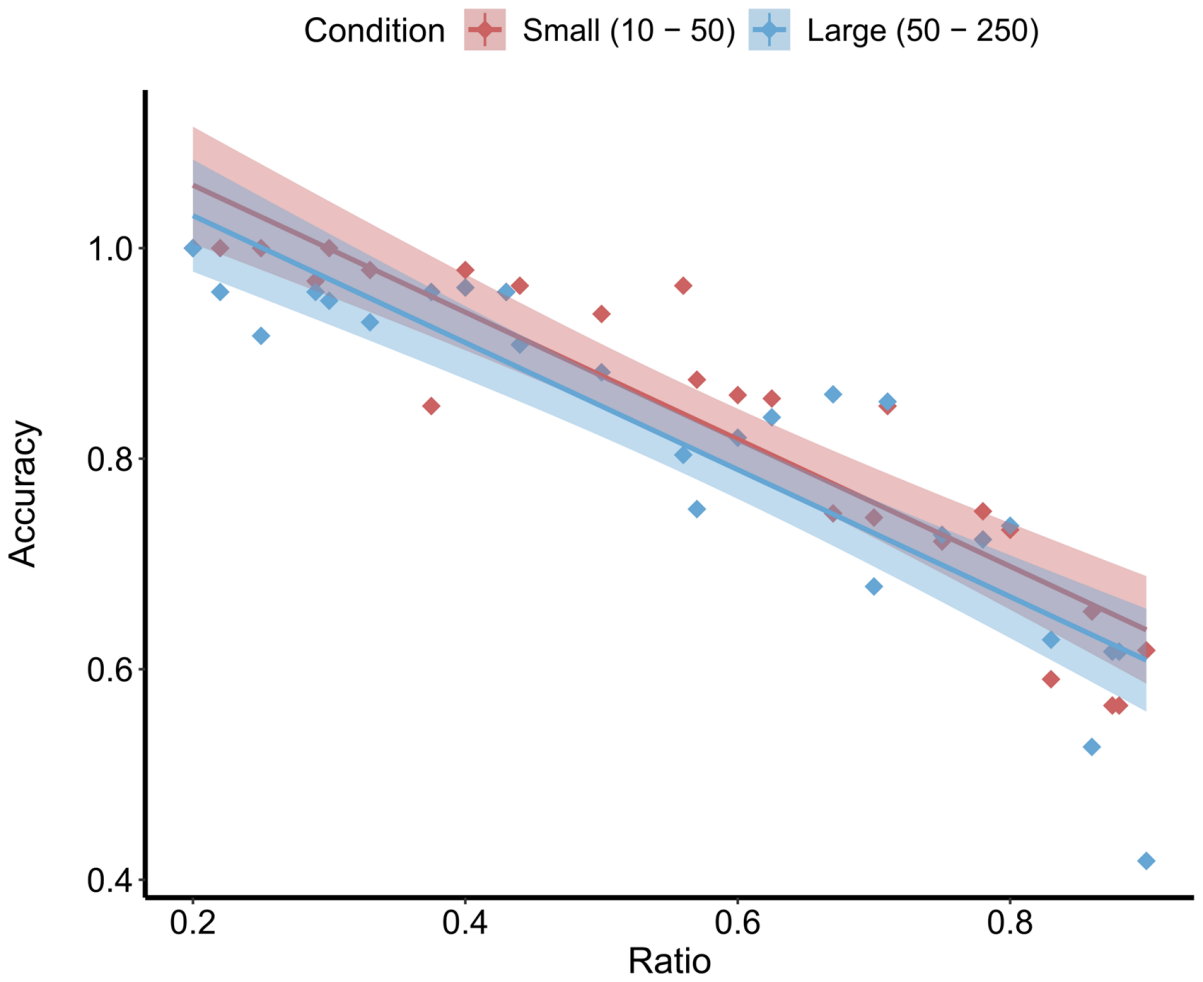
Mean accuracy by ratio on the control trials for both the small and large conditions. Shaded areas represent the 95% confidence intervals.

Participants’ accuracy on the control trials of the magnitude comparison task (no adaptation) was significantly above chance (*M* = 0.785, *SD* = 0.038), *t*(7) = 21.03, *p* < 0.001, *d* = 7.50. A repeated-measures ANOVA with a between-subject factor of stimulus condition (small or large) and a within-subject factor of numerical ratio (between the test and probe stimuli [smaller value / larger value]; range: 0.20–1.0) as predictors of accuracy yielded a significant main effect of ratio, *F*(1, 48) = 223.88, *p* < 0.001, $\eta_{p}^{2}\;$ = 0.82 (see Figure 2). As predicted by Weber’s law, this effect indicates that as the ratio between the test and probe stimuli increased, accuracy decreased. There was no main effect of stimulus condition on accuracy, *F*(1, 48) = 2.54, *p* = 0.117, suggesting that the small and large stimulus conditions were equally difficult. Moreover, there was no stimulus condition by ratio interaction, *F*(1, 48) = 0.00, *p* = 0.998, suggesting that the effect of ratio did not differ as a function of stimulus range.

To analyze the effect of adaptation on performance, for each participant, we calculated the proportion of trials in which the participant chose the test stimulus (adapted location) as more numerous than the probe stimulus (non-adapted location), for each pair of stimulus values (*n* = 81/stimulus condition; 9 test values × 9 probe values) and each task (control and adaptation).

To test whether number is best explained by opponent or multichannel coding, we used a linear mixed effects model, with fixed effects of task (control and adaptation), stimulus condition (small and large), and the numerical ratio between test and probe, and random effect of subject. If number is coded by a multichannel system, then there should be a significant interaction between task and condition, such that adaptation aftereffects differ across stimulus condition. However, if number is coded by an opponent channel system, then there should be no significant interaction between task and condition, such that the adaptation aftereffect is uniform across stimulus condition. The linear mixed effects model yielded a significant interaction between task and condition, *F*(1, 1,274) = 9.47, *p* = 0.002, $\eta_{p}^{2}\;$ = 0.007, suggesting that the adaptation aftereffect differed across stimulus condition (small and large), as predicted by a multichannel system.

To further evaluate whether this interaction is indicative of repulsive, bidirectional aftereffects (i.e., underestimation in the small condition and overestimation in the large condition), for each pair of stimulus values (*n* = 81 / stimulus condition), we calculated the mean proportion of trials in which participants chose the test stimulus as more numerous, by task and condition. We then calculated the difference in these values between task conditions (adaptation – control), for each stimulus condition. These values (from here on, referred to as “Proportion ‘Choose Test’”) reflect the mean adaptation aftereffect across participants, wherein negative values represent underestimation during adaptation relative to control, and positive values represent overestimation during adaptation relative to control, with a significant difference from zero indicating an adaptation aftereffect.

**Figure 3:**
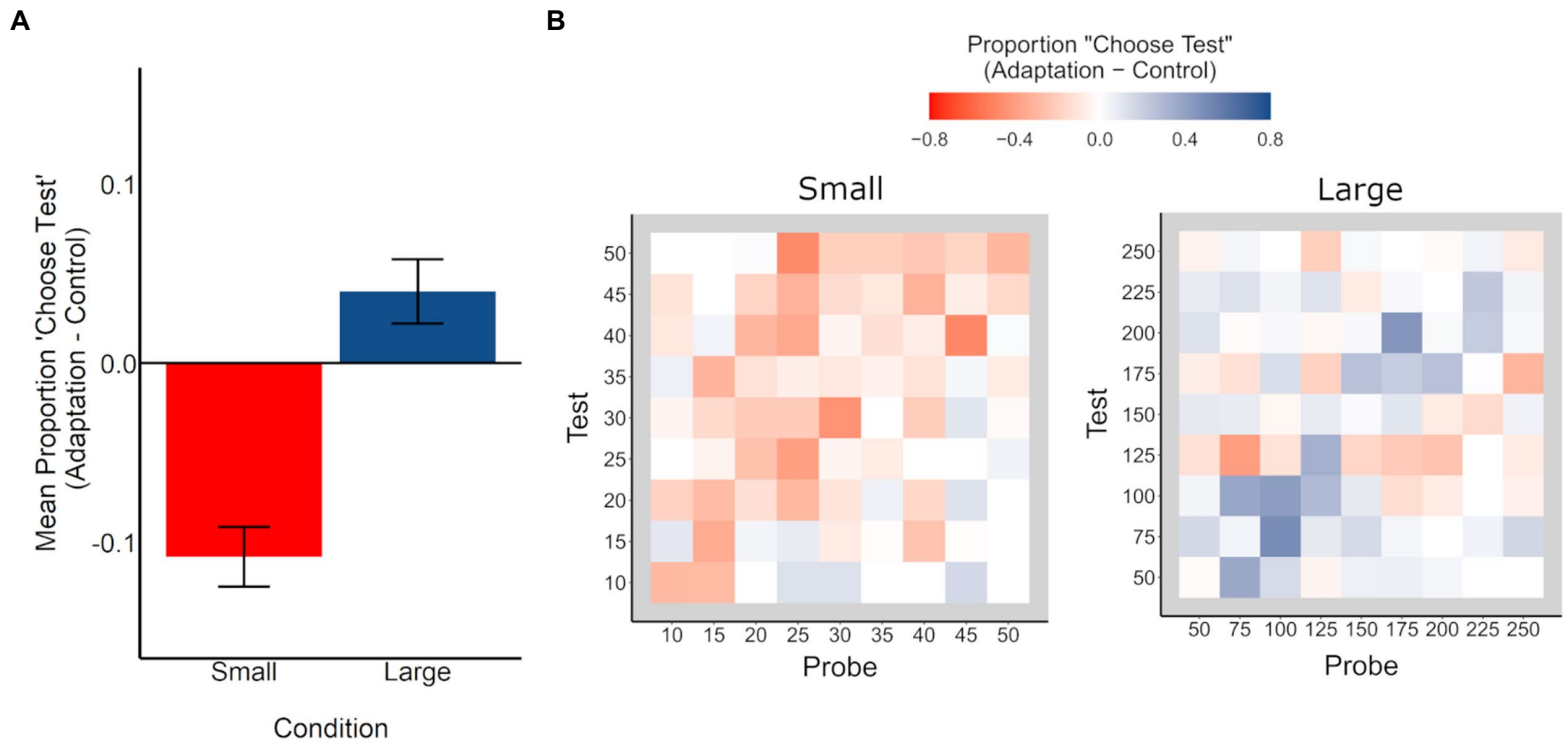
**(A)** Difference in mean proportion of trials in which participants chose the test stimulus as more numerous (adaptation minus control), for small (red) and large (blue) conditions. Negative values reflect underestimation of the test stimulus (adapted location) in the adaptation condition, relative to the control condition, and positive values reflect overestimation of the test stimulus (adapted location) in the adaptation condition, relative to the control condition. Error bars represent +/− 1 SE. **(B)** Difference in mean proportion of trials in which participants chose the test stimulus as more numerous (adaptation minus control) for each test/probe stimulus pair, for small (left) and large (right) conditions. Negative values (red) reflect underestimation of the test stimulus in the adaptation condition relative to the control condition, and positive values (blue) reflect overestimation of the test stimulus in the adaptation condition relative to the control condition. For example, a value of −0.10 suggests that participants chose the test stimulus as more numerous 10% less often in the adaptation condition than in the control condition.

For each stimulus condition, we compared the Proportion “Choose Test” values to zero to evaluate whether there was a significant adaptation effect (see Figure 3). In the small condition, the Proportion “Choose Test” values were significantly smaller than zero, indicating significant underestimation, *t*(80) = 6.52, *p* < 0.001, *d* = 0.72. In the large condition, the Proportion ‘Choose Test’ values were significantly larger than zero, indicating significant overestimation, *t*(80) = 2.23, *p* = 0.029, *d* = 0.25. Moreover, the Proportion ‘Choose Test’ values in the small and large conditions were significantly different from each other, *t*(160) = 6.05, *p* < 0.001, *d* = 0.95, consistent with a bidirectional, as opposed to a unidirectional, aftereffect.

However, could the difference in aftereffects across the small and large conditions instead reflect adaptation of distinct perceptual systems, one for representing number and another for representing texture/density (Anobile et al., 2014; Morgan et al., 2014; Zimmermann, 2018; Pomè et al., 2019)? In other words, if the range of numbers used in the large condition (50–250) tapped both a number system (at relatively smaller values) and a texture/density system (at relatively larger values), then adaptation in the texture/density system could mask an otherwise unidirectional effect of number across conditions. If true, this would predict a significant difference in the direction of the aftereffect between the smaller and larger values in the large condition, such that smaller values should be underestimated and larger numbers should be overestimated (or unaffected). To evaluate this possibility, we directly compared the difference in Proportion “Choose Test” (adaptation – control) between the smaller (75–150) and larger (175–250) values in the large condition. There was no difference between the two ranges, *t*(35) = 0.043, *p* = 0.966, suggesting that texture adaptation at very large values could not account for the difference in aftereffects between small and large conditions.^1^

## Discussion

In the present study, we found that adaptation to an intermediate numerosity (50) resulted in bidirectional aftereffects, such that numbers smaller than 50 were generally underestimated and numbers larger than 50 were generally overestimated. These findings are consistent with a multichannel coding scheme for number, and inconsistent with an opponent channel coding scheme, which predicts unidirectional aftereffects.

Research with nonhuman animals has provided evidence for “labeled-line” coding of number, in which each neuron has a preferred stimulus value (Nieder and Merten, 2007; Ditz and Nieder, 2016). Multichannel coding is consistent with the notion of labeled-line coding. For example, the ventral intraparietal area (VIP) of non-human primates represents number according to a labeled-line system, such that each neuron has a preferred number, where the activation patterns of that neuron are best described by a Gaussian distribution centered on the preferred number (“tuning curve”; Piazza et al., 2004; Viswanathan and Nieder, 2013; Kersey and Cantlon, 2017). Notably, in the present work, we found that adaptation to 50 dots influenced the perception of arrays ranging from 10 to 250 dots, suggesting that adapted channels extend over a very wide range of values. Accordingly, future work will be needed to determine how the wide channels suggested by the present work relate to narrower channels described by work on labeled-line coding of number.

Labeled-line coding is often contrasted with monotonic coding (or summation coding), exhibited by the lateral intraparietal area of non-human primates (LIP; Roitman et al., 2007; Pearson et al., 2010). Monotonic coding would seem consistent with an opponent channel system, wherein all stimulus values are encoded by only two distinct channels (e.g., one that responds maximally to small numerosities and one that responds maximally to large numerosities). Given evidence for both labeled-line and monotonic coding of number in the parietal cortex of non-human primates (Van Opstal, 2007), future work will be needed to determine how these different neural coding systems influence numerical behavior. Specifically, it is not yet clear whether labeled-line and monotonic neural representations of number can be selectively activated and, if so, whether this would result in behavior consistent with multichannel and opponent channel schemes, respectively. If this correspondence were demonstrated, then the present work would suggest that number discrimination, at least in the context of a magnitude comparison task, may be primarily driven by labeled-line coded number representations, potentially in the human homolog of VIP (Harvey et al., 2017).

Contra some previous predictions (Dehaene, 2009; Schwiedrzik et al., 2016), the present findings are inconsistent with opponent channel coding for number. Specifically, Schwiedrzik et al. (2016) examined cross-adaptation between motion and number, such that, following adaptation to leftward or rightward motion, participants exhibited numerical adaptation aftereffects consistent with opponent channel coding. What could account for the differences between our findings and those of Schwiedrzik and colleagues? One possibility is the findings of Schwiedrzik and colleagues may reflect opponent channel coding of motion, not number. Alternatively, another possibility is that the paradigm used by Schwiedrzik and colleagues affected different number representations than those adapted by conventional number adaptation tasks. Specifically, given that dot motion stimuli, like that used by Schwiedrzik and colleagues, preferentially activate LIP (Shadlen and Newsome, 1996; Williams et al., 2003), cross-adaptation between motion and number may have engaged opponent channel representations of number in the human homolog of LIP, rather than multichannel representations of number, perhaps in the human homolog of VIP.

It has been argued that number perception instead reflects the perception of non-numerical magnitudes, such as cumulative area or density (Durgin, 2008; Dakin et al., 2011; Leibovich et al., 2017). And, indeed, there is accumulating evidence that number perception may not be independent of other magnitudes (Aulet and Lourenco, 2021; Lourenco and Aulet, 2022). Could non-numerical dimensions explain the adaptation aftereffects observed in the present work? Because element size and convex hull were held constant in the dot array stimuli used here (see Methods), cumulative area and density were necessarily correlated with number, such that as number increased, cumulative area, and density also increased. As a result, we cannot rule out the influence of these magnitudes on the aftereffects observed. However, and critically, Schwiedrzik et al. (2016) also used stimuli in which number, area, and density were correlated, suggesting that the differences between their and our work cannot be attributed to this aspect of the stimuli. Nevertheless, it is possible that concurrent adaptation to non-numerical magnitudes influenced our results. Accordingly, future work with an adaptation paradigm will be needed to better isolate number from non-numerical magnitudes. Specifically, further investigation into the coding schemes underlying adaptation to non-numerical (continuous) magnitudes will help distinguish numerical from non-numerical processing.

In sum, the present work provides novel evidence that behavioral number adaptation results from a non-opponent, multichannel coding scheme. Our work highlights the power of perceptual adaptation as a tool for characterizing the mechanisms underlying number perception. Furthermore, these findings are consistent with previous neural work on labeled-line coding, suggesting that the adaptation aftereffects observed here may result from a neural coding scheme for number within parietal cortex. Critically, these potential parallels between behavioral and neural coding schemes highlight the power of behavioral paradigms for addressing questions about the neural and computational mechanisms of number perception.

## Data availability statement

The raw data supporting the conclusions of this article will be made available by the authors, without undue reservation.

## Ethics statement

The studies involving human participants were reviewed and approved by Emory University. The participants provided their written informed consent to participate in this study.

## Author contributions

LA and SL designed the experiments and wrote the manuscript. LA collected and analyzed the data. All authors have read and approved the submitted manuscript.

## Funding

This work was supported by a National Institutes of Health (NIH) institutional training grant (T32 HD071845) to LA.

## Conflict of interest

The authors declare that the research was conducted in the absence of any commercial or financial relationships that could be construed as a potential conflict of interest.

## Publisher’s note

All claims expressed in this article are solely those of the authors and do not necessarily represent those of their affiliated organizations, or those of the publisher, the editors and the reviewers. Any product that may be evaluated in this article, or claim that may be made by its manufacturer, is not guaranteed or endorsed by the publisher.

## Footnotes

^1^Given concerns that at large numerosities (e.g., 150–250), stimuli may be encoded by a texture-based, as opposed to number-based, system, we conducted an additional experiment in which we examined adaptation with numbers ranging from 50 to 130 instead of 50 to 250. Across this range, we replicated all major findings presented in the main text, with the difference in Proportion ‘Choose Test’ between adaptation and control conditions being significantly greater than zero, suggesting significant overestimation, *t*(80) = 3.17, *p* = 0.002, *d* = 0.33. Moreover, Proportion ‘Choose Test’ in this range (50–130) was not significantly different from Proportion ‘Choose Test’ in the range reported in the main text (50–250), *t*(160) = 0.56, *p* = 0.57, suggesting that the adaptation aftereffect (overestimation) did not significantly differ across ranges.
